# Delineation of dominant and recessive forms of *LZTR1*‐associated Noonan syndrome

**DOI:** 10.1111/cge.13533

**Published:** 2019-04-03

**Authors:** Alistair T. Pagnamenta, Pamela J. Kaisaki, Fenella Bennett, Emma Burkitt‐Wright, Hilary C. Martin, Matteo P. Ferla, John M. Taylor, Lianne Gompertz, Nayana Lahiri, Katrina Tatton‐Brown, Ruth Newbury‐Ecob, Alex Henderson, Shelagh Joss, Astrid Weber, Jenny Carmichael, Peter D. Turnpenny, Shane McKee, Francesca Forzano, Tazeen Ashraf, Kimberley Bradbury, Deborah Shears, Usha Kini, Anna de Burca, The DDD Study, Edward Blair, Jenny C. Taylor, Helen Stewart

**Affiliations:** ^1^ NIHR Oxford BRC Wellcome Centre for Human Genetics, University of Oxford Oxford UK; ^2^ Manchester Centre for Genomic Medicine St Mary's Hospital, Manchester University Hospitals NHS Foundation Trust, Manchester Academic Health Sciences Centre Manchester UK; ^3^ Wellcome Sanger Institute, Wellcome Genome Campus Cambridge UK; ^4^ Oxford NHS Regional Molecular Genetics Laboratory Oxford University Hospitals NHS Trust Oxford UK; ^5^ South West Thames Regional Genetics Service, St. George's University Hospitals NHS Foundation Trust London UK; ^6^ Department of Clinical Genetics University Hospitals Bristol NHS Trust Bristol UK; ^7^ Northern Genetics Service Newcastle upon Tyne Hospitals NHS Foundation Trust Newcastle upon Tyne UK; ^8^ West of Scotland Regional Genetics Service, Laboratory Medicine Building Queen Elizabeth University Hospital Glasgow UK; ^9^ Department of Clinical Genetics Liverpool Women's NHS Foundation Trust Liverpool UK; ^10^ Oxford Regional Clinical Genetics Service Northampton General Hospital Northampton UK; ^11^ Clinical Genetics Department Royal Devon and Exeter NHS Foundation Trust Exeter UK; ^12^ Northern Ireland Regional Genetics Service Belfast HSC Trust, Belfast City Hospital Belfast UK; ^13^ Clinical Genetics Department Guy's and St Thomas' NHS Foundation Trust London UK; ^14^ Oxford Centre for Genomic Medicine Oxford University Hospitals NHS Foundation Trust Oxford UK

**Keywords:** developmental disorder, exome, *LZTR1*, Noonan syndrome, RAS‐MAPK signalling

## Abstract

Noonan syndrome (NS) is characterised by distinctive facial features, heart defects, variable degrees of intellectual disability and other phenotypic manifestations. Although the mode of inheritance is typically dominant, recent studies indicate *LZTR1* may be associated with both dominant and recessive forms. Seeking to describe the phenotypic characteristics of *LZTR1‐*associated NS, we searched for likely pathogenic variants using two approaches. First, scrutiny of exomes from 9624 patients recruited by the Deciphering Developmental Disorders (DDDs) study uncovered six dominantly‐acting mutations (p.R97L; p.Y136C; p.Y136H, p.N145I, p.S244C; p.G248R) of which five arose de novo, and three patients with compound‐heterozygous variants (p.R210*/p.V579M; p.R210*/p.D531N; c.1149+1G>T/p.R688C). One patient also had biallelic loss‐of‐function mutations in *NEB*, consistent with a composite phenotype. After removing this complex case, analysis of human phenotype ontology terms indicated significant phenotypic similarities (*P* = 0.0005), supporting a causal role for *LZTR1*. Second, targeted sequencing of eight unsolved NS‐like cases identified biallelic *LZTR1* variants in three further subjects (p.W469*/p.Y749C, p.W437*/c.‐38T>A and p.A461D/p.I462T). Our study strengthens the association of *LZTR1* with NS, with de novo mutations clustering around the KT1‐4 domains. Although *LZTR1* variants explain ~0.1% of cases across the DDD cohort, the gene is a relatively common cause of unsolved NS cases where recessive inheritance is suspected.

## 1 INTRODUCTION

Noonan syndrome (NS) is a multisystem condition caused by dysregulation of RAS‐MAPK signalling. Clinical features include a characteristic facial gestalt (broad forehead, hypertelorism, downslanting palpebral fissures, ptosis, small chin), posteriorly‐rotated, low‐set ears, webbing of the neck, widely‐spaced nipples, undescended testes and short stature.^1^, ^2^, ^3^ Skeletal abnormalities can include pectus malformations, spinal deformities and cubitus valgus. Heart defects such as pulmonary stenosis or hypertrophic cardiomyopathy occur in a substantial fraction of patients and the association with intellectual disability has long been recognized.^2^, ^4^, ^5^ The NS phenotype is extremely variable and not all features are observed in all patients. Heterogeneity also exists within families and many mildly affected adults remain undiagnosed until the birth of a more severely affected child. The oft quoted incidence rate of 1/1000‐1/2500 live births^5^ may therefore be an underestimate.

*PTPN11* encodes a protein tyrosine phosphatase implicated in RAS‐MAPK signalling and was the first gene associated with NS, responsible for ~50% of cases.^6^ Several other NS genes with roles in RAS‐MAPK signalling have subsequently been identified. These include *KRAS*, *SOS1*, *RAF1*, *NRAS*, *BRAF*, *RIT1* and *SOS2*.^7^ Despite these gene discovery efforts, a small but significant fraction of cases remain mutation‐negative for known genes.

In contrast to other studies which concentrated on components of RAS‐MAPK signalling,^8^ unbiased exome sequencing of two large Brazilian kindreds identified *LZTR1* as the only gene harbouring rare, predicted‐deleterious variants co‐segregating with NS consistent with an autosomal dominant (AD) mode of inheritance.^9^ Identification of three smaller families with mutations clustering around the same protein‐interaction domains supported *LZTR1* as a novel NS gene (MIM#616564).

Until recently, the mode of inheritance associated with NS has exclusively been AD, with mutations arising de novo or inherited from affected parents. Although sibling recurrence is occasionally seen in families where parents are unaffected, such findings are typically thought to be the result of genetic mosaicism.^10^ Indeed, *PTPN11* mutations undergo positive selection during spermatogenesis.^11^, ^12^ Nevertheless, speculation that an autosomal recessive (AR) form of NS exists, first proposed >25 years ago,^13^ has persisted, supported by the description of NS patients from consanguineous kindreds (MIM#605275; NS2).^14^

A 2018 study involving two large NS families where AR inheritance was considered likely identified overlapping linkage regions each harbouring rare biallelic variants in *LZTR1*. Ten further families were identified, making a total of 23 affected individuals with biallelic alterations.^15^ In this study, we sought to identify patients with *LZTR1*‐associated NS across a well‐defined clinical cohort by performing a systematic analysis of 9624 exomes from the Deciphering Developmental Disorders (DDDs) study. In addition, we used a targeted sequencing approach, selecting clinically diagnosed NS families in whom AR inheritance was considered likely, and performed detailed phenotyping on all affected individuals to help delineate the phenotypic/genotypic ranges of these conditions.

## 2 MATERIALS AND METHODS

### 2.1 Informed consent and exome sequencing

All patients described here (or their parents/legal representative) gave informed consent to participate in this study. The DDD study (www.ddduk.org) focuses on children with undiagnosed developmental disorders and aims to develop translational genomics workflows to feedback potentially diagnostic findings.^16^ Patients were recruited from the 24 Regional Genetics Services in the UK and Republic of Ireland under Research Ethics Committee approval (10/H0305/83; Cambridge South REC, and GEN/284/12; Republic of Ireland REC). Exome sequencing methods have been described previously.^16^ Data analysis involved mapping reads to hs37d5 and calling variants with GATK/CoNVex. De novo variants were called using DeNovoGear. The analysis described here was performed on a data freeze corresponding to 7832 parent‐parent‐child trios and 1792 patient singletons.

### 2.2 Data availability

Data is accessible from the EGA archive (https://ega-archive.org/; EGAS00001000775.)

### 2.3 Variant filtering

Candidate variants in 9624 probands were identified across all genes using the following steps:

1. Minor allele frequency (MAF) was restricted to <0.1% for probands analysed as part of parent‐child trios. This was reduced to <0.01% (ExAC allele count of <5) for dominant variants in singletons. Hemizygous variants had a maximum MAF of <0.1% in trios/singletons and an ExAC hemizygous count = 0. For recessive variants, MAF was required to be <1% in trios/singletons.
2. Annotation using VEP^17^ had to predict the most severe consequence to be a loss‐of‐function or protein‐altering change. Inherited variants predicted benign by PolyPhen2 were excluded. Deletions/duplications had to be >1 Mb.
3. The genotypes and observed inheritance pattern had to be consistent with dominant, recessive or X‐linked modes of inheritance

Candidate variants were then interrogated, looking for specific patients where there was at least one qualifying variant in *LZTR1*. For such cases, we obtained vcf files, filtered lists of all candidate variants in that individual, information about previously reported SNV/indels/CNVs and detailed clinical data.

### 2.4 Analysis of human phenotype ontology terms

Clinical information was collected using human phenotype ontology (HPO), a standardized vocabulary of phenotypic abnormalities (http://human-phenotype-ontology.github.io/). The significance of phenotypic similarity between *LZTR1*‐positive patients was estimated by comparing similarity of HPO terms between the patients of interest to that between randomly selected patients from the diverse DDD cohort, as described.^18^, ^19^

### 2.5 Sanger sequencing, allele‐specific PCR and relationship confirmation

PCR amplification of *LZTR1* was performed as described in Table S1. An additional amplicon was included to capture intron 16 where variants can lead to retention of an alternative exon.^15^ Following enzymatic purification, Sanger sequencing was performed using BigDye (version 3.1) and the ABI 3730XL (Applied Biosystems, Foster City, California).

Where parental samples were unavailable, compound‐heterozygous variants were phased by allele‐specific PCR whereby the 3′‐base of the first primer was complementary to either the wild‐type or mutant allele (Table S1). Sanger sequencing was then performed to determine the sequence at the second locus. A similar method was used to phase a de novo variant where the closest informative SNP was too distant to be phased by Illumina read‐pairs (Table S1).

For one family where the patient underwent exome sequencing as a singleton, maternity/paternity were confirmed by genotyping nine short tandem repeat (STR) loci using the AuthentiFiler PCR Amplification Kit (ThermoFisher Scientific, Waltham, Massachusetts).

## 3 RESULTS

### 3.1 Four likely‐pathogenic de novo LZTR1 variants identified in patient‐parent trios

**Figure 1:**
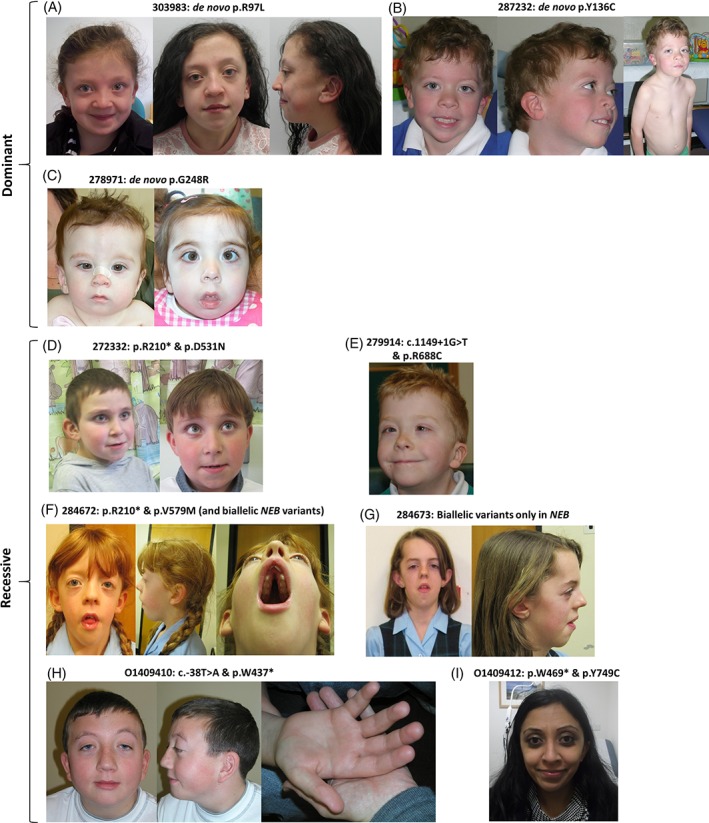
Clinical images showing Noonan‐like features in patients where consent was obtained. A, Patient 303983 aged 7.5 and 14 years showing hypertelorism and low‐set posteriorly rotated ears. B, Patient 287232 aged 5 years showing low‐set ears, pointed chin and pectus excavatum. C, Patient 278971 shown at 11 months and 3.7 years showing epicanthic folds and depressed nasal bridge and D, Patient 272332 aged 8.6 years and 10.2 years with interrupted eyebrow, long palpebral fissures, low‐set posteriorly rotated ears, thin top lip and narrow chin. E, Patient 279914 aged 6.8 years showing a convergent squint, ptosis, low‐set posteriorly rotated ears and wide neck. F, Patient 284672 aged 8 years showing long face, hypertelorism, proptosis, downslanting palpebral fissures, retrognathia, macrodontia and a high, narrow palate. G, Patient 284673 (284672's elder sister) aged 11 years—while both siblings had biallelic *NEB* variants, the elder sister did not have both *LZTR1* variants and so is shown for comparison. Although both sisters have myopathy and bilateral ptosis, the younger sibling has a stronger NS gestalt. H, Patient O1409410 aged 12 years showing down‐slanting palpebral fissures, palpebral ptosis, low‐set posteriorly rotated ears and fetal finger‐tip pads. I, Patient O1409412 aged 28 years showing a narrow nasal root and broad nasal tip

Among 7832 parent‐parent‐child trios, exome sequencing uncovered five patients with de novo missense mutations in *LZTR1*, all called with high confidence (posterior probability for the de novo configuration, pp_dnm >0.9). Of these, four clustered around kelch domains KT1‐4 (codons 79‐285, UniProt Q8N653), had CADD scores of 26‐34 and thus were deemed likely pathogenic. More genetic information and clinical characteristics of these patients are in Table 1, Table S2 and Figure 1A‐C. The 5th de novo variant (c.2074T>G; p.F692V) lies outside KT1‐4 and a hemizygous *RPS6KA3* variant provides a better explanation for the phenotype (https://decipher.sanger.ac.uk/patient/266615).

**Table 1:** Details of LZTR1 variants identified, analysis of HPO term similarity and Face2Gene rankings

Screening method	Mode of inheritance	DECIPHER or local ID	Chr22 position	Ref/alt alleles	Variant annotation	dbSNP150	CADD	gnomAD	Trio genotype	HPO terms	HPO term similarity *P‐*value			Face2Gene ranking of Noonan syndrome (Figure S1)
Exome sequencing as part of DDD study	Autosomal dominant	269172	21 342 304	T>C	c.406T>C; p.Y136H	NA	25	NA	1/0/0^b^	Abnormal echocardiogram, Abnormal spatial orientation of the cardiac segments, Abnormality of the abdomen, Abnormality of the atrioventricular valves, Abnormality of the heart valves, Coarctation of aorta, Hepatomegaly, Large for gestational age, Malrotation of small bowel, Malformation of the heart and great vessels	NA ‐ from singleton analysis so phenotype used for prioritisation			#1 (medium) aged 3 mo #2 (medium) aged 8 y (data not shown)
		271777	21 342 332	A>T	c.434A>T; p.N145I	NA	29	NA	1/1/0^b^	Abnormal facial shape, Drooling, Global developmental delay, Hyperreflexia, Hypertonia, Long face, Myopathic facies, Narrow forehead, Periventricular leukomalacia, Specific learning disability				NA
		303983	21 340 156	G>T	c.290G>T; p.R97L	NA	34	NA	1/0/0 (pp_dnm = 1)	Atria septal defect, Downslanted palpebral fissures, Epicanthus, Hypertrophic cardiomyopathy, Short stature, Ventricular septal defect	0.0478			#1 (high) aged 7.5 y #1 (high) aged 14 y
		287232	21 342 305	A>G	c.407A>G; p.Y136C	NA	26	NA	1/0/0 (pp_dnm = 1)	2–3 toe syndactyly, Barrel‐shaped chest, Cryptorchidism, Delayed speech and language development, Depressed nasal bridge, Generalised hypotonia, Low‐set posteriorly rotated ears, Motor delay, Unilateral ptosis, Wide intermamillary distance				#1 (high) aged 5 y
		274799	21 344 754	C>G	c.731C>G; p.S244C	NA	29	NA	1/0/0 (pp_dnm = 1)	Cafe‐au‐lait spot, Hypermetropia, Low‐set posteriorly rotated ears, Pectus carinatum, Short stature, Single transverse palmar crease, Strabismus, Webbed neck				#2 (low) aged 7 y (data not shown)
		278971	21 344 765	G>A	c.742G>A; p.G248R	rs869320686	34	1/245964^a^ (AF = 5/34)	1/0/0 (pp_dnm = 0.93)	Depressed nasal bridge, Epicanthus, Microcephaly, Preauricular pit, Prominent metopic ridge, Severe global developmental delay				#1 (high) aged 11 mo #13 (low) aged 3 y 8 mo
	Autosomal recessive	272332	21 348 534	G>A	c.1591G>A; p.D531N	rs138615487	34	7/230636	1/1/0	Autistic behavior, Global developmental delay, Hypertrophic cardiomyopathy, Long palpebral fissure, Mitral valve prolapse, Pes planus, Prominent fingertip pads, Seizures	0.0629			#10 (low) aged 8.5 y #3 (low) aged 10 y
			21 343 948	C>T	c.628C>T; p.R210*	rs150419186	40	19/276182	1/0/1					
		279914	21 346 659	G>T	c.1149+1G>T	rs767191322	23	1/241846	1/1/0	Bilateral ptosis, Blue irides, Downslanted palpebral fissures, Hyperacusis, Joint hypermobility, Low‐set posteriorly rotated ears, Pectus carinatum, Proportionate short stature				#1 (medium) aged 6 y 10 mo
			21 350 154	C>T	c.2062C>T; p.R688C	rs587777178	35	14/275020	1/0/1					
		284672	21 343 948	C>T	c.628C>T; p.R210*	rs150419186	40	19/276182	1/1/0	Bifid uvula, Bilateral ptosis, Downslanted palpebral fissures, Generalised joint laxity, Hearing impairment, High palate, Hypertelorism, Long face, Macrodontia, Myopathy, Pectus excavatum, Pointed chin, Renal duplication, Retrognathia				#1 (medium) aged 8 y
			21 348 966	G>A	c.1735G>A; p.V579M	rs765416902	32	4/245336	1/0/1					
Sanger sequencing		O1409410	21 348 001	G>A	c.1311G>A; p.W437*	rs770933647	39	NA	1/1/0	NA–HPO terms not collected. Patients recruited due to suspected autosomal recessive Noonan syndrome.				#1 (high) aged 12 y
			21 336 623	T>A	c.‐38T>A	NA	10	NA	1/0/1					
		O1409412	21 348 266	G>A	c.1407G>A; p.W469*	rs777243508	40	42/272718	1/0/1^c^					#1 (medium) aged 28 y
			21 351 011	A>G	c.2246A>G; p.Y749C	rs755260815	28	1/246114	1/1/0^c^					
		O1504902	21 348 241	C>A	c.1382C>A; p.A461D	NA	31	NA	1/1/0^c^					NA
			21 348 244	T>C	c.1385T>C; p.I462T	rs147684991	26	5/274258	1/0/1^c^					

Chromosome positions use hs37d5 and annotations are based on NM_006767.3. Individual order for trio genotype is proband/mother/father, with 1 = heterozygous and 0 = homozygous reference. pp_dnm is the posterior probability for the de novo configuration.

a Indicates non‐PASS variant, possibly due to mosaicism. AF indicates allelic fraction observed for variant.

b Parental genotypes are from Sanger sequencing. The mother of individual 271777 was mildly affected (see Supplementary note 1).

c Variants initially shown to be *in trans* by allele‐specific PCR.

**Figure 2:**
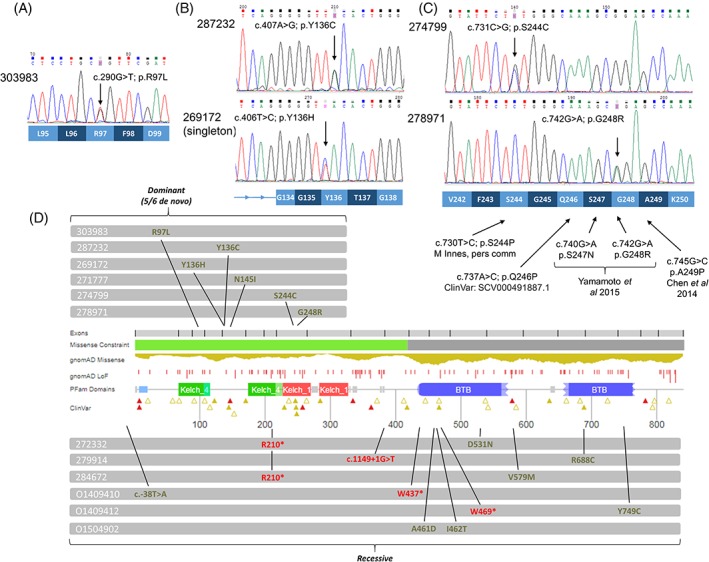
Sanger validation of de novo mutations and distribution of autosomal dominant and recessive variants across *LZTR1*. A, Validation data for the c.290G>T; p.R97L variant. B, Validation data for two de novo variants that disrupt Tyr136. C, Validation data for de novo variants disrupting Ser244/Gly248 alongside other variants reported to be associated with Noonan syndrome (NS). D, Distribution of variants identified in this study along the LZTR1 protein. Figure adapted from protein summary view at DECIPHER (https://decipher.sanger.ac.uk/gene/LZTR1#overview/protein‐info). Likely loss of function variants shown in red, missense and 5’‐UTR variants shown in green

Patient 303983 harbored a de novo c.290G>T; p.R97L variant (NM_006767.3) within the KT1 domain of LZTR1 (Figure 2A). She was recruited to DDD with suspected NS but *PTPN11* and several other NS genes were mutation‐negative. Clinical features include a typical NS gestalt (Figures 1A and S1A), hypertrophic cardiomyopathy, a ventricular septal defect and short stature. She is planning to have surgery on her spine due to kyphosis and anterior fusion of T9‐12 intervertebral discs.

Patient 287232 harbored a de novo c.407A>G; p.Y136C variant within KT2 (Figure 2B). Hypotonic at birth, this boy has a classical NS gestalt, with coarse facial features, left‐sided congenital ptosis, low‐set ears, pointed chin and pectus excavatum (Figures 1B and S1B). He has mild pulmonary valve stenosis, short stature and developmental delay (particularly affecting motor skills).

Patient 274799 harbored a de novo c.731C>G; p.S244C variant within KT4 (Figure 2C). This boy had low‐set, posteriorly rotated ears, webbing of the neck and pectus carinatum, leading to a strong clinical suspicion of NS. Mutations in *PTPN11* and several other NS genes had previously been excluded.

Patient 278971 harbored a nearby de novo variant (c.742G>A; p.G248R, Figure 2C), also within KT4. Here, the de novo configuration was less certain (pp_dnm = 0.93) but later confirmed by Sanger sequencing of patient/parental DNA. Unlike the other de novo variants identified, p.G248R was reported in gnomAD as a singleton (Table 1) and in two independent NS families.^9^, ^20^ Patient 278971's most significant clinical issues relate to perinatal asphyxia. She had seizures in the neonatal period, which resolved for a period of time but later recurred. Aged 8 years, she weighs only 16 kg. She attends a special school, cannot read or write, walks only with an aid/frame and has extremely limited speech. She had successful mitral valve surgery 3 years ago to control regurgitation. She now has marked micrognathia such that intubation is always an ordeal. Although she had/has features within the NS spectrum such as low‐set ears, neck webbing, pectus excavatum and mild cubitus valgus, facial features are now atypical for NS (Figures 1C and S1C). No additional clinically relevant genetic variants were identified and so we speculate that her phenotype may have been complicated by antenatal/perinatal insults which have resulted in severe intellectual disability and growth restriction.

### 3.2 Analysis of singleton exomes identifies additional de novo/inherited variants

A qualifying heterozygous variant in *LZTR1* was identified in 25/1792 singleton exomes (Table S3). Of these, two were missense changes involving KT1‐4, both absent from gnomAD. The first of these was a heterozygous c.406T>C; p.Y136H alteration in female patient 269172 with significant cardiac abnormalities. The variant notably disrupts the same codon as the de novo c.407A>G; p.Y136C. We subsequently validated the variant (Figure 2B) and confirmed its absence from parental DNAs, consistent with it having arisen de novo. In contrast, six other rare candidate variants observed in 269172 were inherited from unaffected parents (Table S4). Together with data from nine STRs, these results helped confirm maternity/paternity. This patient's main clinical issues are cardiac and include severe pre‐ductal coarctation, bicuspid aortic valve, “parachute” mitral valve and left atrial isomerism. Although there is a mild NS‐like gestalt, this was more prominent earlier in development.

The second notable variant in a singleton was c.434A>T; p.N145I in patient 271777 (III‐4). His phenotype included global developmental delay, learning difficulties, drooling, hyperreflexia, hypertonia, periventricular leukomalacia and an abnormal/myopathic long facial shape; in childhood it was suggested that he may have NS. The same variant was detected independently in a first cousin (III‐1) using exome sequencing and a virtual RASopathy gene‐panel. III‐1 has mild pulmonary stenosis, mild learning difficulties, a webbed neck and facial features reminiscent of NS. A fuller description of this family and results of segregation testing in other mildly affected family members is presented in Supporting Information, Supplementary note 1.

### 3.3 Phasing of de novo mutations

In the four cases where likely pathogenic de novo variants (p.R97L, p.Y136C, p.S244C, p.G248R) were identified by trio exome sequencing, informative intronic SNPs were identified 68 bp‐136 bp away (Table S5). For singleton patient 269172, the closest identifiable informative SNP was >5 kb away, so phasing of the p.Y136C mutation was performed using allele‐specific PCR (Table S1). In all five cases, the de novo variant had arisen on the paternal chromosome (Table S5).

### 3.4 Recessive LZTR1 cases and a “blended” phenotype of NS and nemaline myopathy

Three unrelated individuals harbouring biallelic variants in *LZTR1* were found. These individuals were also identified in a global analysis of the DDD cohort that sought to identify novel disease genes in addition to quantifying the overall contribution of recessive variants to developmental disorders.^19^ The variants identified were all rare, with gnomAD MAFs of 1/241846 to 19/276182 and CADD scores of 23 to 40 (Table 1). Additional information for these patients is shown in Table S6 and Figure 1D‐F.

Patient 272332 inherited a c.1591G>A; p.D531N variant *in trans* with c.628C>T; p.R210*. An unaffected brother only harbored the p.D531N variant. Using the ACMG criteria for assessing pathogenicity,^21^ p.D531N is ranked as a variant of uncertain significance (VUS, Table S7). Cardiac features included hypertrophic cardiomyopathy and mitral valve prolapse, requiring surgery; these led to NS being considered as a potential diagnosis. Prior testing of *PTPN11* was negative. Although this male patient had low‐set, posteriorly rotated ears, a narrow chin, foetal fingertip pads and short stature, the facial features of this patient were considered to be more suggestive of Kabuki syndrome (Figures 1D and S1D). He has moderate to severe intellectual disability, autism spectrum disorder and tonic‐clonic seizures which recently returned, aged 14 years.

Patient 279914 inherited a splice donor site variant (c.1149+1G>T) and a c.2062C>T; p.R688C. Although prior testing for *PTPN11* and other NS genes was negative, the patient had a presumed NS diagnosis, with notable features including blue irides, downslanting palpebral fissures, hypertelorism, low‐set posteriorly rotated ears (Figures 1E and S1E), pectus carinatum and short stature.

Patient 284672 was recruited to the DDD study together with her elder affected sister (284673). Loeys‐Dietz syndrome, myotonic dystrophy and facioscapulohumeral muscular dystrophy were proposed as potential diagnoses but genetic testing for these conditions did not detect any pathogenic variants (Table S6). Although nemaline myopathy was considered another possibility, prior analysis of *NEB* (NM_001271208.1) had been limited to testing for a founder deletion involving exon55.^22^ Exome sequencing identified rare biallelic variants in *NEB* in both siblings: c.78+1G>A *in trans* with a novel c.21489‐21493dupGACTG; p.A7165fs*84. In addition to these *NEB* variants, the younger sister (284672) was found to harbour the c.628C>T; p.R210* variant in *LZTR1 in trans* with a c.1735G>A; p.V579M VUS. The older sister had not inherited both *LZTR1* variants. Our interpretation of data from this family is therefore that while both girls have nemaline myopathy, the younger sibling has a more complex “blended” phenotype due to additional biallelic variants in *LZTR1*. Consistent with this hypothesis, we note that features specific to the younger sibling include hypertelorism, pointed chin, webbed neck, a broad chest, pectus excavatum, mitral‐valve abnormalities and abnormal nuchal translucency scan results during pregnancy. Comparison of facial features is also consistent with the younger sibling having a more NS‐like gestalt (Figure 1F‐G, Figure S1F‐G).

### 3.5 Phenotype comparisons using unbiased HPO terms

To help support that the *LZTR1* variants were of clinical relevance, we tested whether the patients identified above were more similar than expected by chance. Comparison of HPO terms for the four patients in whom trio exome analysis had uncovered de novo dominant mutations in KT1‐4 domains of *LZTR1* showed significant phenotypic similarity (Table 1; *P* = 0.0478). Singleton patient 269172 was excluded from this analysis as prioritisation of this case from a group of singletons had included review of phenotype information. Comparison of the three patients with compound‐heterozygous variants also showed a degree of similarity, although this did not reach a formal level of significance (*P* = 0.0629), as reported previously.^19^ Grouping the AD and AR cases together increased the significance levels of phenotypic similarity (*P* = 0.0019). Finally, we removed the AR patient (284672) with biallelic mutations in *NEB* (as well as *LZTR1*) as we believed this patient to have a “blended” phenotype, with features such as myopathy and ptosis more likely due to *NEB*. This comparison of the remaining six cases further increased levels of similarity (*P* = 0.0005).

### 3.6 Biallelic LZTR1 variants in 3/8 patients with a clinical diagnosis of NS

Our second mutation detection strategy involved Sanger sequencing of *LZTR1* in seven unrelated patients with a diagnosis of NS and one with the clinically overlapping Costello syndrome. These patients had all tested negative for multiple NS genes. Aiming to enrich the cohort for AR forms of NS, six of these cases had been selected either due to documented consanguinity or because of the presence of affected siblings.

Patient O1409410 is a male with classic NS facial features (Figures 1H and S1H). In this individual, we identified compound‐heterozygous variants c.1311G>A; p.W437* and c.‐38 T>A (Table 1); the latter introduces an alternative ATG codon out‐of‐frame with the canonical start site. Such features in mRNA, also known as upstream open reading frames (uORFs), can cause widespread protein expression changes in humans,^23^ in some cases resulting in disease.^24^, ^25^ We therefore speculated that c.‐38T>A might alter the amount of wild‐type *LZTR1* protein. Using a 100 bp sequence spanning the canonical start site and the c.‐38T>A locus we ran ATGpr/TISminer on wild‐type and mutant sequences. This analysis suggested that, while both sites have an equally strong identity with the [A/G]XXATGG Kozak consensus sequence, the novel ATG could have a higher reliability in initiating translation (Supplementary note 2). A reporter assay using a dual luciferase strategy was performed, as described.^23^, ^25^ The ratio of renilla to firefly luciferase was consistently reduced to 77% to 85% for the mutant 5’‐UTR in comparison to the WT (Supplementary note 2). AR inheritance had been suspected in this family as a male sibling (despite normal early scans) developed polyhydramnios at 20 weeks gestation. Severe fetal hydrops ensued and an emergency cesarean section was performed at 38 weeks due to reduced fetal movements. The baby died shortly after delivery. A postmortem showed an increased heart mass of 24.6 g (normal 16.4 ± 5.7 g) but no structural abnormality. Histology of skeletal and cardiac muscle showed an excess of muscle spindles, noteworthy given reports of such anomalies in Costello syndrome.^26^ Sanger sequencing indicated that this individual harbored both variants, while an unaffected sister did not.

Patient O1409412, a female with milder NS‐like features (Figures 1I and S1I) and severe hypertrophic cardiomyopathy, harbored compound‐heterozygous variants; c.1407G>A; p.W469* and a c.2246A>G; p.Y749C VUS. Allele‐specific PCR (Table S1) was used to indicate these variants lay *in trans*, later confirmed by testing parental DNAs. Again, AR inheritance had been suspected in this family as a male sibling born at 34/40 weeks had hypertrophic cardiomyopathy and died at 18 months. DNA for this individual was unavailable for testing.

Patient O1504902 harbored two rare missense VUSs 3 bp apart (c.1382C>A; p.A461D and c.1385T>C; p.I462T). Allele‐specific PCR confirmed that the two variants lay *in trans* (Figure S2). This girl was born to unaffected non‐consanguineous parents, has cardiac hypertrophy and a NS‐like appearance. No pathogenic variants were detected in several other NS‐related genes. A younger unaffected sister has not been tested. Sanger sequencing of *LZTR1* in the other five patients did not reveal any significant variants.

### 3.7 Clinical comparison and Face2Gene analysis

Review of available photographs indicates that a depressed nasal bridge in young children with narrow nasal root and broad nasal tip in older children are characteristic features of *LZTR1*‐associated NS. In AD cases it appears that the face may elongate with age (Figure 1A,C). Analysis of photos using the Face2Gene tool (www.face2gene.com) showed that for 8/10 patients where photographs were obtained, NS ranked highest for at least one age (Table 1). For 4/8 of these matches, there was a high degree of similarity to NS (Figure S1).

Based on a patient described by Johnston et al^15^ and a subsequent case report, it was suggested that the phenotype seen in *LZTR1*‐associated NS may sometimes include growth hormone (GH) deficiency.^27^ The case‐series reported here supports this hypothesis as 287232 had GH deficiency, with a GH stimulation test showing only borderline responses and no rare variants were detected in 40 other genes linked to GH deficiency.

In the original *LZTR1* report, cardiac abnormalities mainly involved pulmonary stenosis.^9^ Only 287232, O1504902 and III‐1 have pulmonary stenosis in this case‐series, with the most common cardiac abnormality being hypertrophic cardiomyopathy, reported in 3/10 AD and 4/6 AR cases, respectively (Table S2 and S6).

## 4 DISCUSSION

In the present study, a systematic analysis of exome data from a large cohort of patients with developmental disorders identified *LZTR1* mutations in ~0.1% (9/9624) of cases. For 8 individuals the *LZTR1* variant(s) are likely causative and in one further case we hypothesise *LZTR1* is contributing to a “blended” phenotype. This figure may be an underestimate, given that non‐coding variants such as c.‐38T>A are poorly captured by exome sequencing. Patients where deletions of <1 Mb are playing a role would also have been overlooked due to the filtering pipeline employed.

Yamamoto et al^9^ suggested AD‐acting mutations are most likely to occur in the KT1‐4 protein interaction domains, in particular KT4. Consistent with this, we identified de novo mutations at residues p.S244 and p.G248 (Figure 2C). The two de novo mutations involving p.Y136 (Figure 2B) may represent a novel hotspot. Further studies describing other de novo disease‐causing mutations will help refine the regions implicated in the AD form of NS. A recent structural analysis of LZTR1 indicates that AD‐acting variants typically lie on the top surface of a six‐blade propeller‐like structure.^28^ Most de novo variants identified here fit with that pattern, however p.R97L and the familial p.N145I are buried toward the side of the propeller structure; we suspect these may abrogate phosphorylation (Supplementary note 3, www.matteoferla.com/LZTR1.html). In the AR form, compound‐heterozygosity often involves a LoF allele *in trans* with a presumed hypomorphic variant, with mutations typically spread across the gene (Figure 2D). The exception to this rule was O1504902 who harbored missense variants at adjacent codons (Figure S2).

The clinical team responsible for O1504902 could not find (as of April 2018) any accredited UK laboratories offering *LZTR1* testing as part of a NS service, despite the fact *LZTR1* was first associated with NS >3 years ago.^9^ This highlights the variable lag time for inclusion of new genes on panels and hence the advantages of using an exome sequencing approach. Together with the 4/50 incidence seen in a Brazilian cohort,^9^ the 3/8 detection rate obtained from our targeted sequencing approach emphasises the importance of updating NS/RASopathy panels to include this gene. This is further supported by the ClinGen Expert Panel's recent assessment of 19 genes associated with various RASopathies where the evidence for *LZTR1* was categorised as strong.^7^ Virtual gene‐panels (curated gene‐lists used to filter variants detected by exome/WGS analysis) are more readily updatable. For instance, the RASopathy panel in PanelApp (https://panelapp.genomicsengland.co.uk/panels/48/) revised the mode of inheritance for *LZTR1* from monoallelic to monoallelic/biallelic just 6 weeks after publication.^15^

NS is part of a clinical spectrum of conditions which include Noonan‐like disorders due to mutations in *SHOC2, PPP1CB* and *CBL*, Costello syndrome caused by *HRAS* mutations and cardiofaciocutaneous syndrome caused by disruption of *BRAF, MAP2K1* and *MAP2K2*. These disorders all result from dysregulation of RAS‐MAPK signalling so have collectively been termed the “RASopathies”. *LZTR1* has only recently been functionally linked to RAS‐MAPK signalling,^20^, ^28^, ^29^, ^30^ which explains why its role in NS long went unrecognised. For instance, the gene was absent from a list of 789 RAS–ERK pathway genes prioritised by Chen et al^8^ such that the pathogenicity of p.R237Q/p.A249P identified in that study was only realised subsequently.^9^ Recent work by Motta et al indicates that missense alterations associated with AR inheritance typically influence protein synthesis/stability or subcellular localization. In contrast, mutations associated with the AD form are expressed normally but enhance EGF‐dependent ERK1/2 phosphorylation.^28^ Functional studies such as these are especially important for the missense alterations described here where pathogenicity has not been conclusively established (Table S7, Supplementary note 4).

The c.‐38T>A variant we identified leads to a uORF. Several examples exist in the literature of disease‐causing variants in 5‐‘UTRs and reporter gene assays such as the one used here are commonly used to confirm the effect of such variants upon translation. Examples include a de novo c.‐107G>A variant in *SLC2A1* found in a patient with glucose transporter deficiency syndrome and c.‐263C>A/c.‐255G>A variants in *TWIST1* in patients with Saethre‐Chotzen syndrome.^24^, ^25^

Using a combination of parent‐child exomes and allele‐specific PCR showed that 5/5 de novo mutations reported here occurred on the paternal chromosome. Although a larger case‐series is required to reach significance, these results are notable given mutations in *PTPN11* and other RAS‐MAPK genes can influence spermatogonial selection and predominantly occur on the paternal chromosome.^31^ A 2004 study phased 14 de novo mutations in *PTPN11* and found all originated on the paternal haplotype.^11^ The mean paternal age in that *PTPN11*‐positive cohort was 35.6 years, 2.2 years above that for the *PTPN11*‐negative cases and 6.1 years older than the population average. Increased paternal age at conception and a similar bias in the parental origin of de novo *HRAS* mutations have been documented in patients with Costello syndrome.^32^, ^33^ For the five cases with de novo *LZTR1* mutations described here, paternal ages at childbirth were also elevated (mean = 35.8, Table S5) compared with the average across this DDD datafreeze (mean = 32.6).

A recent study focussing on fetal malformations detected in utero identified a case of non‐immune hydrops fetalis with a homozygous variant in *LZTR1*.^34^ Fetal hydrops was also observed in 4 non‐liveborn siblings described by Johnston et al.^15^ Together with siblings of O1409410/O1409412, these results indicate that *LZTR1* mutations can result in a much more severe form of disease. Further studies should aim to uncover the reasons for this extreme variability and whether such lethal presentations of disease can also be associated with the AD form of *LZTR1*‐associated NS.

Combining our results with those described in the literature,^9^, ^15^, ^20^, ^27^ hypertrophic cardiomyopathy was reported in 5/26 of individuals with AD‐acting mutations but 19/26 of those harbouring biallelic variants (Tables S2 and S6). A systematic analysis of cardiac involvement in larger clinical cohorts of patients with *LZTR1* mutations is warranted to confirm whether this bias is reproducible.

In conclusion, our study strengthens the association of *LZTR1* with AD/AR forms of NS. In the dominant condition, mutations cluster around the KT1‐4 domains. In the AR form, compound‐heterozygosity often involves a LoF allele *in trans* with a presumed hypomorphic variant. Although *LZTR1* mutations explain only ~0.1% of cases in the DDD study, the gene is a notable cause of unsolved NS cases, especially where recessive inheritance is suspected.

## CONFLICT OF INTEREST

The authors have no conflict of interest to report.

## Supporting information

Appendix S1 Supporting Information.
